# Vitamin D Insufficiency Prior to Paediatric Liver Transplantation Is Associated with Early T-Cell Mediated Rejection

**DOI:** 10.3390/children8070612

**Published:** 2021-07-20

**Authors:** Nathalie M. Rock, Elisa Anghileri, Vladimir L. Cousin, Laetitia-Marie Petit, Valérie A. McLin

**Affiliations:** 1 Swiss Paediatric Liver Center, Paediatric Gastroenterology, Hepatology and Nutrition Unit, Division of Paediatric Specialties, Department of Paediatrics, Gynecology and Obstetrics, Geneva University Hospitals, University of Geneva, 6, Rue Willy-Donzé, 1211 Geneva, Switzerland; elisa.anghileri@gmail.com (E.A.); Vladimir.Cousin@hcuge.ch (V.L.C.); LaetitiaMarie.Petit@hcuge.ch (L.-M.P.); valerie.mclin@hcuge.ch (V.A.M.); 2 School for General Medicine, Polis Lombardia, via Taramelli 12, 20124 Milano, Italy; 3 Division of Intensive Care, Department of Acute Medicine, Geneva University Hospitals, University of Geneva, Rue Gabriel-Perret Gentil 4, 1211 Geneva, Switzerland

**Keywords:** vitamin D, liver transplantation, T-cell mediated rejection, paediatric, plasma parathyroid hormone

## Abstract

**Objectives**: T-cell mediated rejection (TCMR) can compromise long-term liver allograft survival. The immunomodulatory properties of vitamin D are increasingly recognized. We investigated whether perturbations in vitamin D metabolism prior to LT may predispose to TCMR in a representative cohort of paediatric LT recipients. **Methods**: In this retrospective single-center study of children who underwent liver transplantation between 2005 and 2017, we collected serum 25(OH) vitamin D levels and other parameters related to vitamin D metabolism. Post-transplant variables were collected from medical records during the first year following LT. **Results**: Eighty-two patients were included. Twenty-six (32%) developed TCMR, 52 (65%) presented at least one event of 25(OH) D insufficiency during the year before the transplant, while 23 (32%) had at least one documented elevated plasma parathyroid hormone level. Forty-six patients benefited from nutritional support (56%). The development of TCMR was associated with vitamin D insufficiency pre-LT (*p* = 0.01). No significant correlations were identified between PTH levels and incidence of TCMR. The association was stronger in patients transplanted for cholestatic diseases (*p* = 0.004). **Conclusions**: Vitamin D insufficiency before a liver transplant may be associated with TCMR during the first year post-LT. These findings warrant further investigation.

## 1. Introduction

Liver transplantation (LT) is the standard of care for children with end-stage liver disease [1]. Cholestasis is the most frequent indication for paediatric LT and is frequently associated with profound fat-soluble vitamin malabsorption, and consequent vitamin D insufficiency [2]. Further, paediatric LT may differ from adult LT by its comparatively higher frequency of T-cell mediated rejection (TCMR) [3,4].

The immunomodulatory role of vitamin D has been increasingly highlighted. Vitamin D supplementation and vitamin D sufficiency have been reported to be associated with rejection after solid organ transplantation [5,6,7,8,9,10,11]. In adults, the relationship between vitamin D deficiency and the onset of autoimmune diseases, such as multiple sclerosis or rheumatoid arthritis, is increasingly documented [12].

**Figure 1:**
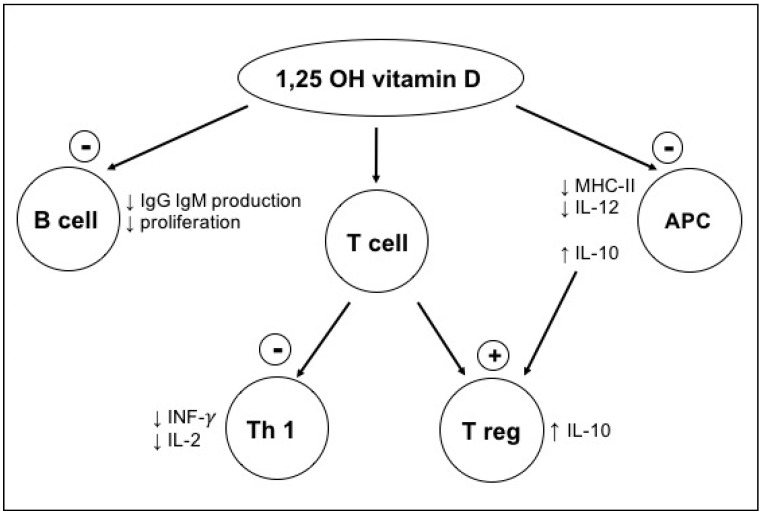
Immunomodulatory role of 1,25 (OH) vitamin D. Figure adapted from [18].

Vitamin D supplementation has been shown to increase the numbers of tolerogenic circulating T-regulatory (Treg) cells in the peripheral blood of adult LT recipients [13]. At a cellular level, the vitamin D receptor (VDR) has been shown to be highly expressed in activated CD4+ and CD8+ T-cells, as well as in B cells, neutrophils, and monocytes, all of which are involved in the alloimmune response. Vitamin D signalling via the VDR contributes to the downregulation of both MHC-II surface expression and IL-12 synthesis in APCs. It also inhibits the synthesis of pro-inflammatory cytokines IL-2 and interferon gamma. Finally, signalling via VDR contributes to IL-10 production, which shifts T-cell polarization from a Th1 to Treg phenotype, and Tregs in turn inhibit both Th1 and antigen presenting cells (APC) [14,15,16,17,18]. All in all, these effects result in an adaptive immunity inhibition (Figure 1) necessary to protect against allograft rejection. 

In light of these new data on the immunomodulatory role of vitamin D and the fact that many children are vitamin D insufficient at transplant, we postulated that vitamin D would be an easily modifiable factor to minimize the risk of TCMR in paediatric liver transplant recipients. Therefore, the aim of our study was to analyse the relationship between pre transplant vitamin D metabolism and the occurrence of TCMR. 

## 2. Methods

This was a single-centre, observational retrospective study of children who underwent LT between January 2005 and December 2017. Patients aged 0–18 years old who had received a pre-transplant vitamin D assessment were included in the pre LT and post LT analysis. Those who died or were lost to follow up in the year post LT were excluded from the study. Data were collected from electronic medical records at the following time-points: (1) Pre-transplant assessment; (2) Follow up visits during the year before transplant; and (3) Post-transplant visits at 3, 6, 9, and 12 months post LT. The study was approved by the institutional ethics committee and all participating patients consented (CER11-010R).

### 2.1. Pre LT Assessment

The following recipient parameters were collected at the pre LT assessment visit: nutritional support, vitamin D supplementation, indication for transplantation (primary disease, which included cholestatic and non-cholestatic disease), height and height Z-score, weight and weight Z-score, calcium and phosphate blood levels, and kidney function. At every visit during the year before transplant, and if available, the following laboratory values were collected: 25 (OH) vitamin D, intact plasma parathyroid hormone (PTH). 

All patients underwent meticulous nutritional assessment, which included clinical parameters such as weight, height, and head circumference measurements. Height Z-score and weight Z-score were calculated using the calculator of the National Health and Nutrition Examination Survey NHANES. A dedicated dietician calculated daily dietary intake for every patient. The goal of this assessment was to ensure adequate nutritional support defined as 100% of the recommended calcium intake for age, 100–150% of the caloric needs for age, and to cover the increased energy expenditure of chronic liver disease. If these caloric or calcium needs were not met with per oral feeding, supplemental nutrition was administered and defined as partial or total administration of caloric needs by nasogastric tube or parenteral nutrition. Moreover, patients with insufficient 25(OH) D levels, despite nutritional support, received additional supplementation. 

### 2.2. Post LT Events

T-cell mediated rejection was defined as a Banff score ≥ 3 in clinically indicated liver biopsies, evaluated by two senior histopathologists [17,19]. Tacrolimus trough levels and target range were recorded. The time to the first TCMR episode and number of TCMR episodes within the first year after transplantation were analysed. Tacrolimus trough level goals were as follows: 10–12 ng/mL post-operative (PO) days 0–30, 8–10 ng/mL PO days 31–180, 6–8 ng/mL PO days 180–365 and 4–6 ng/mL thereafter. A trough level goal was considered achieved if tacrolimus was >8 ng/mL at 3 months. 

### 2.3. Definitions

25 (OH) vitamin D was measured using the LaRoche vitamin D total assay. Vitamin D insufficiency was determined if 25 (OH) D was < 30 ng/mL (75 nmol/L) [20]. Vitamin D supplementation was defined as present/absent based on discharge summaries. An elevated PTH level was defined as intact PTH > 6.8 pmol/L. Any abnormal value of PTH or vitamin D occurring during the year before transplant was considered as a relevant event. 

Cholestatic liver disease was defined as a pre-LT diagnosis of biliary atresia, Alagille syndrome, progressive familial intrahepatic cholestasis, and unknown causes of chronic cholestasis. Other indications for transplant included metabolic and neoplastic diseases, acute liver failure, portal hypertension, and other rare indications. 

For the purpose of this study, kidney function was considered abnormal if GFR was < 60 mL/min/1.73 m^2^. Below this level renal hydroxylation of 25 (OH) D in 1,25 (OH) D is impaired, leading to secondary hyperparathyroidism [21].

Seasonality or ethnicity were not considered, because we estimated that the impact of liver disease would far outweigh that of possible sun exposure in a group of patients who were mostly indoors pre LT.

### 2.4. Statistics

Continuous data were expressed as median with interquartile range (IQR). Categorical variables were expressed as numbers and percentages. Comparative analyses were performed using the Fisher exact test or a Chi^2^ test depending on numbers for frequencies comparison and the Mann-Whitney test for continuous variables. We used cumulative incidence curves with the Kaplan-Meier method for survival analysis and compared it with the log-rank test. In case of a significant log-rank test, we performed a Cox regression model. Due to the relatively small number of patients, we performed a limited number of univariate and no multivariate analysis. Analyses were performed using Stata 14.1 (StataCorp, College Station, TX, USA). *p* Values ≤ 0.05 were considered statistically significant.

## 3. Results

### 3.1. Patient Characteristics

Ninety-two (92) patients aged 0–16 years who underwent liver transplantation between 2005 and 2017 qualified for the study. Ten patients were excluded: five died within the first year after LT, three owing to missing data, and two were lost to follow up. Eighty-two (82) patients were included in the study (Table 1). The median time between the relevant event (vitamin D insufficiency or elevated PTH) and transplant was 83 days (IQR: 40–146 days).

### 3.2. Immune Suppression Protocol

The primary immunosuppression protocol was tacrolimus-based (81/82) with basiliximab induction for all patients except for 10. Fifty-five patients transplanted in 2009 or later (67%) received per protocol steroids during the first 3 months post LT.

### 3.3. TCMR

Twenty-six (26; 32%) developed T-cell mediated rejection (Table 2). Fifty-three percent (14/26) of children who developed TCMR received steroids as part of their primary immunosuppression protocol. The frequency of TCMR was similar in the groups with and without steroids (*p* = 0.13). In case of rejection, patients received high dose steroids (5 mg/kg) weaned over 5 weeks. There was no episode of steroid-resistant TCMR.

### 3.4. 25(OH) Vitamin D Insufficiency

**Figure 2:**
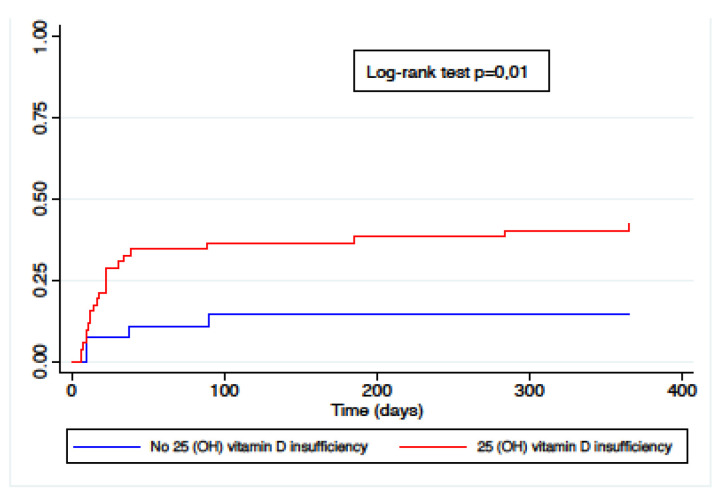
Cumulative incidence of TCMR by pre LT vitamin D status.

**Figure 3:**
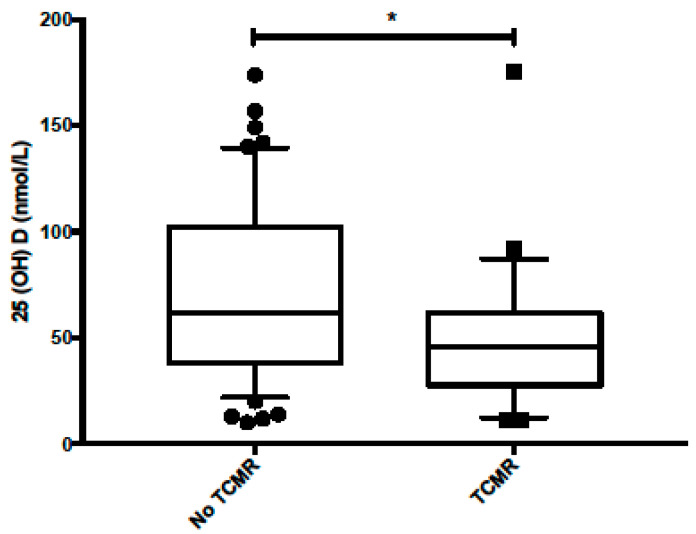
Comparative plot of 25(OH) levels between no-TCMR and TCMR group. * *p* = 0.03.

Sixty-five percent of the study population presented one episode of vitamin D insufficiency during the year pre LT (52/80), 42% of which (22/52) developed TCMR. Of the 28 patients without pre LT vitamin D insufficiency, 14% (4/28) developed at least one episode of TCMR. Eighty-four percent (69/82) were supplemented with vitamin D according to clinical condition and 25(OH) D status. The cumulative incidence of TCMR was significantly higher in patients with any episode of pre LT vitamin D insufficiency, as shown in Figure 2, with a hazard ratio of 3.5 (CI 1.2–10.1, *p* = 0.02). The comparative plot of 25(OH) levels between the TCMR group and no-TCMR group is presented in Figure 3. The median vitamin D levels were 62 nmol/L in the no-TCMR group [IQR 39–100] compared to 45 nmol/L [IQR 30–61] (*p* = 0.03).

### 3.5. Elevated Plasma PTH Levels and Nutritional Status

A pre LT elevated plasma PTH level was not associated with a higher incidence of TCMR (*p* = 0.28). No association between pre LT height, weight z-score and TCMR was identified (Table 2).

### 3.6. Cholestatic Disease Subgroup

Fifty-four (54/82) (66%) patients underwent LT for cholestatic disease. Of these, 48/54 (88%) had elevated plasma levels of conjugated bilirubin at the time of the pre-transplant evaluation. As shown in Table 3, the association between TCMR occurrence and 25(OH) vitamin D insufficiency was stronger in the subgroup with primary cholestatic disease compared to the whole cohort (*p* = 0.004).

## 4. Discussion

Vitamin D insufficiency is an easily modifiable factor before liver transplant if monitored and supplemented. Because of the immunomodulatory role of vitamin D, we hypothesized that pre LT 25(OH) D levels may predispose to TCMR. In this representative cohort of paediatric liver transplant recipients, including both cholestatic and non-cholestatic diseases, pre LT vitamin D insufficiency affected 65% of patients. 25(OH) D insufficiency was associated with TCMR prevalence and cumulative incidence during the first year post LT. The association was notable among patients with cholestatic disease.

Recently, others have explored the possible link between vitamin D status and alloimmunity. The evidence in kidney, lung, heart, and liver transplantation suggests that vitamin D may protect from TCMR [5,22]. In kidney transplant recipients, recent data suggest a positive correlation between vitamin D supplementation and the absence of post-transplant autoimmune diseases or acute cellular rejection [6,7,9,13]. Ban et al. observed the immunologic outcomes after kidney transplantation in 174 patients. A high serum vitamin D level at baseline was independently associated with a low incidence of acute rejection [11]. In adult LT recipients, several studies suggest the association between low vitamin D and TCMR. In a cohort of 141 adult patients after liver transplantation, vitamin D deficiency was identified as an independent risk factor for TCMR, with a lower incidence in the group receiving vitamin D supplementation. In this group, Treg cells and T memory cells were increased compared to the group without supplementation [10]. Lower levels of serum vitamin D at the time of LT were associated with more severe TCMR (BANFF > 6) in an adult cohort [13]. Likewise, early vitamin D supplementation after liver transplantation was associated with an absence of acute rejection in a group of adult recipients [6]. In a paediatric study analysing a cohort of 528 patients, vitamin D insufficiency in the post LT period was associated with lower survival after LT, and vitamin D supplementation *after* transplantation with a lower risk of TCMR [23]. Our study adds to this body of literature by suggesting that in children, in whom cholestasis and fat-soluble, vitamin malabsorption is the norm prior to LT, evidence of vitamin D insufficiency *before* transplantation may be associated with the risk of TCMR. This novel finding is also interesting because paediatric recipients are typically expected to experience more TCMR than adults and vitamin D levels are modifiable both before and after LT [3,4].

The association between TCMR and 25(OH) D insufficiency was more significant in the group of patients who underwent LT for cholestatic disease, possibly skewing the data for the whole cohort. This is despite the fact that 25(OH) vitamin D insufficiency was not more frequent in the cholestatic group than in the non-cholestatic group. A plausible explanation is a longer exposure to 25(OH) insufficiency owing to malabsorption prior to LT [24].

It is encouraging to have identified a very simple, modifiable factor that could impact long-term graft and patient outcomes. A practical approach is to measure 25(OH) vitamin D quarterly before transplant and actively supplement patients to reach a target serum level of 30 ng/mL (75 nmol/L). Cholestatic patients have increased vitamin D needs. Due to fat malabsorption, enteral supplementation is often not sufficient to reach the desired serum levels of 25(OH) vitamin D. This is why in many centres parenteral supplementation by intramuscular injection of 25(OH) vitamin D is recommended when bilirubin is elevated (>60–80 mM/l) [24,25,26]. In this cohort, the effect of vitamin D supplementation on TCMR is difficult to analyse. Indeed, detailed doses of vitamin D supplementation and the route of administration were not available, owing in part to the fact the patients were managed together with other centres [27,28,29,30,31].

This finding adds to the growing clinical focus on pre-transplant nutritional management of paediatric LT candidates [32]. It appears that not only does pre LT nutritional management reduce early post-operative complications and risk, but it may also have more long-term, far-reaching effects on allograft inflammation or tolerance [27,28,29,30,31].

These compelling findings need further validation and are marred by some of the limitations of the study which spans years during which both pre- and post-transplantation practices have evolved.

Other important limitations of the study are the small sample size, which dictated only univariate analyses, the retrospective design, and the heterogeneity of primary diagnoses, which included both cholestatic and non-cholestatic diseases.

## 5. Conclusions

The present study suggests that vitamin D insufficiency prior to paediatric LT may be associated with a cumulative risk of TCMR. These findings add to the growing body of literature highlighting the immunomodulatory role of vitamin D metabolism in immunity and in solid organ transplantation. They are ripe for validation and auspicious for a simple intervention.

## Figures and Tables

**Table 1:** Demographics, baseline characteristics, pre-transplant, transplant and post-transplant factors.

	Number (Percentage) or Median [Q25, Q75]
**Pre LT data**	***N* = 82**
Gender (male)	49 (60)
Age at transplant (months)	57 [10,108]
**LT data**	
**Indication for LT**	
Cholestatic disease	54 (66)
Metabolic disease	11 (13)
Neoplastic disease	6 (7)
Acute liver failure	5 (6)
Others	4 (5)
25(OH)-D insufficiency *	52 (65)
Vitamin D supplementation	69 (84)
Elevated plasma PTH *	23 (32)
Nutritional support	46 (56)
Weight Z score	−0.94 [−1.9,0.85]
Height Z score	−0.49 [−1.2,0.7]
CKD GFR <60 mL/min	11 (13)
**Post LT events**	
TCMR	26 (32)
Unique TCMR episode	24 (29)
BANFF score	
3–5	10 (12)
6–7	12 (14)
8–9	3 (4)
Undetermined	1 (1)

The data are presented as the number (percentage) or median [interquartile range], as appropriate. *N* = number. Pre LT/post LT: pre/post liver transplantation. 25(OH)D: 25 (OH) vitamin D. Q: interquartile range. PTH: plasma parathyroid hormone. CKD: chronic kidney disease. GFR: glomerular filtration rate. TCMR: T-cell mediated rejection. BANFF score: 3 borderline, 4–5 mild, 6–7 moderate, 8–9 severe. * 25(OH)-D and PTH missing for 2 patients: *N* = 80.

**Table 2:** Comparison between group with and without TCMR.

	No TCMR = 56 (68)	TCMR = 26 (32)	*p*-Value
**Pre LT data**			
Cholestatic disease	37 (66)	17 (65)	1.0
Age at LT (months)	30 [10,115]	18 [11,51]	0.74
Pre LT weight Z-score	−0.93 [−1.8,0.06]	−1.03[−2.13,0.05]	0.6
Pre LT height Z-score	−0.53[−1.53,0.7]	−0.23 [−1.2,0.6]	0.66
Nutritional support	31 (55)	15 (58)	1.0
PELD	7 [–4,18]	11[5,24]	0.1
MELD	14 [10,24]	18 [10,24]	0.37
**Metabolism D vitamin**			
25(OH)-D insufficiency *	30 (53)	22 (85)	**0.01**
Elevated plasma PTH *	13 (23)	9 (35)	0.28
Vitamin D supplementation	46 (82)	23 (88)	0.53
Nutritional support	31 (55)	15 (58)	1.0
CKD GFR < 60 (mL/min)	10 (17)	1 (4)	0.16
**Immediate post LT data**			
Intubation time	1.5 [1,5]	3 [1,5]	0.6
Days hospitalization	34 [26,44]	39 [30,77]	0.1
**Immune suppression post LT**			
With steroids	41 (73)	14 (53)	0.13
Tacrolimus > 8 ng/mL at 3 months	34 (60)	19 (73)	0.3
Complications post LT **			
CMV PCR positive	13 (23)	3 (11)	0.25
EBV PCR positive	16 (29)	2 (8)	0.08

The data are presented as the number (percentage) or median [interquartile range], as appropriate. Total number of patients within each group is the reference to calculate the percentage. (No TCMR /56; TCMR /26). N = number. TCMR: T-cell mediated rejection. Pre LT/post LT: pre/post liver transplantation. 25(OH)D: 25 (OH) vitamin D. PTH: plasma parathyroid hormone. CKD: chronic kidney disease. * 25(OH)-D and PTH missing for 2 patients: N=80. ** For TCMR group, positive PCR were considered if performed before rejection.

**Table 3:** TCMR in patients with cholestatic disease *N* = 54.

	No-TCMR = 37 (68)	TCMR = 17(31)	*p*-Value
**Pre LT data**			
Age at LT (month)	15 [9,84]	13 [10,42]	0.99
Pre LT weight Z-score	−1.3 [1.9,2.2]	−1.2 [2.2,0.33]	0.75
Pre LT height Z-score	−0.6 [−1.5,0.67]	−0.1[−1.2,0.7]	0.5
Nutritional support	20 (54)	12 (70)	0.37
Bilirubin pre transplant > 17 umol/L	32 (86)	16 (94)	0.65
**Vitamin D metabolism**			
25(OH)-D insufficiency *	19 (51)	16 (94)	**0.004**
Elevated plasma PTH *	11 (30)	7 (41)	0.37
Vitamin D supplementation	35 (97)	17 (100)	1.0

The data are presented as the number (percentage) or median [interquartile range], as appropriate. Total number of patients within each group is the reference to calculate the percentage. (No TCMR /37; TCMR /17). *N* = number. Pre LT: pre liver transplantation. PTH: plasma parathyroid hormone. TCMR: T-cell mediated rejection. * 25(OH)-D and PTH missing for 2 patients: *N* = 53.

## Data Availability

Data are available upon request to the corresponding author.
